# Left ventricular‐only fusion pacing versus cardiac resynchronization therapy in heart failure patients: A randomized controlled trial

**DOI:** 10.1002/clc.23616

**Published:** 2021-08-03

**Authors:** Yangang Su, Wei Hua, Farong Shen, Jiangang Zou, Baopeng Tang, Keping Chen, Yixiu Liang, Lang He, Xiaohong Zhou, Xue Zhang, Hongyang Lu, Shu Zhang

**Affiliations:** ^1^ Department of Cardiology Zhongshan Hospital of Fudan University, Shanghai Institute of Cardiovascular Diseases, National Clinical Research Center for Interventional Medicine Shanghai 200032 China; ^2^ Arrhythmia Center, State Key Laboratory of Cardiovascular Disease Fuwai Hospital, National Center for Cardiovascular Diseases, Chinese Academy of Medical Sciences and Peking Union Medical College Beijing China; ^3^ Department of Cardiology Zhejiang Greentown Cardiovascular Hospital Hangzhou China; ^4^ Department of Cardiology The First Affiliated Hospital of Nanjing Medical University Nanjing China; ^5^ Department of Pacing and Electrophysiology The First Affiliated Hospital of Xinjiang Medical University Urumqi China; ^6^ Cardiac Rhythm Management Medtronic plc Mounds View Minnesota USA; ^7^ Cardiac Rhythm Management Medtronic Technology Center, Medtronic (Shanghai) Ltd Shanghai China

**Keywords:** AdaptivCRT, cardiac resynchronization therapy, left ventricular fusion pacing

## Abstract

**Background:**

It is unclear whether clinical benefits of cardiac resynchronization can be achieved by pacing only the left ventricle.

**Hypothesis:**

We aimed to compare the effect of a novel adaptive left ventricular‐only fusion pacing (LVP) on ventricular function with conventional biventricular pacing (BVP) in cardiac resynchronization therapy (CRT) indicated patients.

**Methods:**

This prospective, randomized, multicenter study enrolled CRT‐indicated patients with PR interval ≤ 200 ms who were randomized in the adaptive LVP group (using the AdaptivCRT™ algorithm with intentional non‐capture right ventricular pacing) or the echocardiography‐optimized BVP group. Cardiac function and echocardiography were evaluated at baseline and follow‐ups. CRT super response was defined as two‐fold or more increase of left ventricular ejection fraction (LVEF) or final LVEF >45%, and LV end‐systolic volume (LVESV) decrease >15%, and New York Heart Association (NYHA) class improved by at least one level.

**Results:**

Sixty‐three patients were enrolled in the study (LVP = 34 vs. BVP = 29). At 6‐month follow‐up, significant improvements in LVEF, LVESV, and NYHA class were observed in both groups. The CRT super response rate was significantly higher in patients with high‐percentage adaptive LV‐only pacing in LVP group (68.4%) than in BVP group (36.4%, p = .04).

**Conclusions:**

Adaptive LV‐only pacing was comparable to BVP in improving cardiac function and clinical condition in CRT‐indicated patients. This finding raises the possibility that an adaptive LVP algorithm with appropriate right ventricular sensing to fuse with intrinsic right ventricular activation in a two‐lead (right atrium and left ventricle) device may provide clinical benefit in a subset of CRT patients with intact atrioventricular conduction.

## 1 BACKGROUND

Cardiac resynchronization therapy (CRT) improves cardiac function and clinical outcomes in patients with symptomatic heart failure with a decreased left ventricular ejection fraction (LVEF) and prolonged QRS duration (QRSd), preferentially left bundle branch block (LBBB).^1^, ^2^ The underlying mechanism is the resynchronization of left ventricular (LV) and right ventricular (RV) activation. However, CRT pacing modality does not consider the utilization of intrinsic RV conduction and dynamic changes in atrioventricular (AV) conduction. Recently, LV‐only pacing is proposed as an alternative approach to achieve resynchronization by the fusion of LV pacing with intrinsic RV conduction.^3^ Although acute and short‐term investigations suggested comparable benefits of LV‐only pacing and conventional biventricular pacing (BVP),^4^, ^5^, ^6^ varying AV conduction may still lead to electrical dyssynchrony during various daily activities or due to the changes in disease state over a longer period of time. Therefore, an adaptive algorithm is developed to optimize the fusion by continuously adjusting LV pacing timing to leverage intrinsic RV conduction and achieve dynamic and more physiological pacing. The purpose of this study is to assess mid‐term cardiac function and clinical outcomes during adaptive LV‐only fusion pacing (LVP) in comparison with conventional BVP in a selected group of CRT‐indicated patients with intact AV conduction.

## 2 METHODS

### 2.1 Study population

Patients were recruited in five centers. Inclusion criteria were (1) aged between 18 and 80 years old; (2) NYHA class II or III after guideline‐directed medical treatment for at least 3 months; (3) LVEF ≤35%; and (4) sinus rhythm, LBBB pattern with QRSd ≥150 ms and intrinsic PR interval ≤200 ms. Exclusion criteria included severe hepatic or renal dysfunction, persistent atrial fibrillation, AV block, upgrade from a pacemaker or defibrillator device, valvular heart disease, pregnancy, inability to give informed consent or to perform the follow‐up assessments. The establishment of this study was approved by the Ethical Committees of Shanghai Zhongshan Hospital, Fuwai Hospital, Zhejiang Greentown Cardiovascular Hospital, The First Affiliated Hospital of Nanjing Medical University, and The First Affiliated Hospital of Xinjiang Medical University. All procedures followed the ethical standards of the Ethical Committees and in line with the 1964 Helsinki Declaration. Written informed consent of clinical information collection and follow up was acquired from each patient at admission.

### 2.2 Study design

This study is a prospective, multicenter, randomized, controlled clinical trial. Consented patients who fulfilled inclusion and exclusion criteria were enrolled and randomly assigned to LVP or BVP group. Randomization was performed among all patients. Centralized random sequence was generated from natural number with odd or even number standing for LVP or BVP group, then site staff would assign each patient to a corresponding group and intervention per random sequence. Ethics committee approvals were obtained in all study sites. Written informed consent was obtained from each patient. The study was registered at http://www.clinicaltrials.gov under the identifier NCT03071978.

All enrolled patients received CRT devices featured with the AdaptivCRT™ algorithm (Medtronic Inc., Minneapolis, MN) according to clinical demands. The AdaptivCRT algorithm^7^, ^8^, ^9^, ^10^ is a novel pacing algorithm for CRT by dynamically optimizing the AV and interventricular (VV) delays minute‐by‐minute based on the electrical conduction intervals. Furthermore, for patients with normal AV conduction, the AdaptivCRT algorithm recruits the intrinsic conduction and avoids providing RV pacing. For patients in LVP group, AdaptivCRT pacing mode was enabled after implantation to provide maximum fusion of LV pacing with intrinsic RV activation, while pacing through RV lead was functionally turned off (set as the minimal pacing parameters to make sure of non‐capture RV pacing) to achieve the LV‐only pacing. For patients in BVP group, AdaptivCRT was disabled after implantation, then AV and VV delays were optimized before discharge using echocardiographic evaluation with the method described by Gorcsan et al.^11^ The pacing mode settings of enrolled patients will be determined by physician according to the clinical status after completing the 6‐month follow‐up and exiting this study.

### 2.3 Clinical and cardiac function assessments

Assessments of cardiac function and clinical outcomes were conducted at 3‐ and 6‐month follow‐ups. The primary endpoint was the improvement in LVEF; the secondary endpoints included (1) NYHA class and 6 min walking distance (6MWD); (2) QRSd; and (3) LV end‐systolic volume (LVESV), LV end‐diastolic volume (LVEDV), LV end‐systolic dimension (LVESD), and LV end‐diastolic dimension (LVEDD) by echocardiographic assessments.

Echocardiographic measurements were performed with a commercially available system (Vingmed Vivid 7; GE Vingmed, Milwaukee, WI). Echocardiographic data were recorded for at least three consecutive cardiac cycles. LVEF, LVESV, and LVEDV were measured by Simpson's biplane method from the apical four‐chamber view. Parameters were analyzed with a consistent protocol in a core laboratory. CRT response was defined as an absolute increase of LVEF >10% or a relative decrease of LVESV >15% or NYHA class improved by at least one level at 6‐month follow‐up compared to baseline value. In addition, CRT super response^12^ was defined as the composite score of a two‐fold or more increase of LVEF or a final LVEF >45%, and LVESV decrease >15%, and NYHA class improved by at least one level at 6 months.

### 2.4 Statistical analysis

Continuous variables were presented as means ± standard deviation, and categorical variables as numbers and proportions. Continuous variables were compared between baseline and 3‐/6‐month follow‐up using paired Student's *t*‐test or Wilcoxon signed‐rank test. For comparisons of continuous variables between the LVP and BVP groups, independent Student's *t*‐test or Mann–Whitney *U* test was applied. Categorical variables were compared using the chi‐square test or Fisher's exact test. Statistical significance was defined as a p < .05. All analyses were performed with SPSS software (version 22; SPSS, Chicago, IL).

## 3 RESULTS

### 3.1 Patient characteristics

**FIGURE 1:**
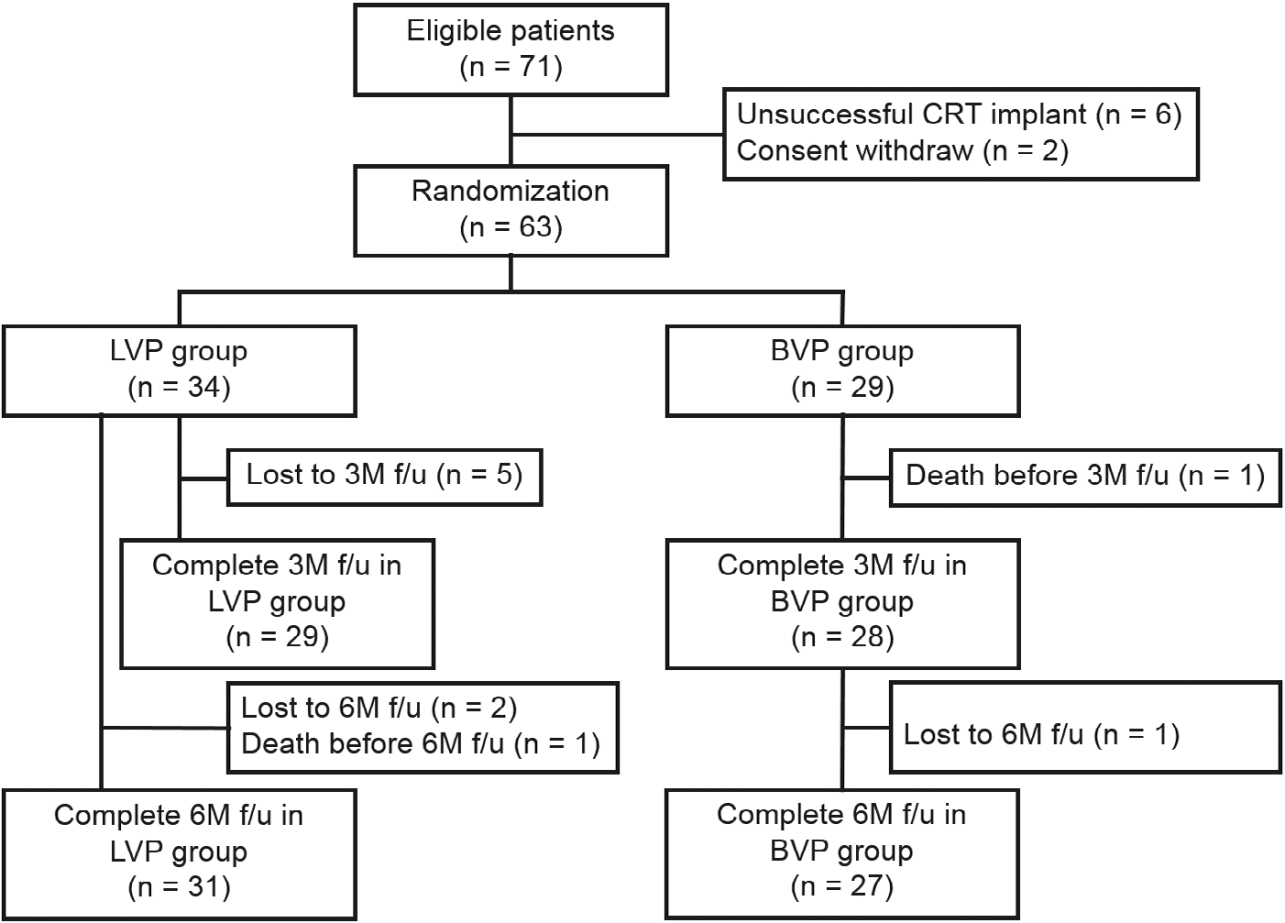
Patient flow chart. 3 M f/u, 3‐month follow‐up; 6 M f/u, 6‐month follow‐up

Sixty‐three consecutive patients were prospectively enrolled from April 2017 to June 2018 and randomly assigned to LVP group (*n* = 34) or echocardiography‐optimized BVP group (*n* = 29). Study follow‐ups were completed in Jan 2019. The numbers of loss of follow‐up at 6 months were three in LVP group and two in BVP group (Figure 1), which included one mortality in each group. The cause of the two deaths was lung cancer. Characteristics of the study population at baseline are summarized in Table 1. At baseline, patients (aged 63 ± 11 years, 71.4% male) had symptomatic heart failure (NYHA class 2.7 ± 0.5), decreased LVEF (27% ± 7%), increased LVESV (168.4 ± 67.2 ml), prolonged QRSd (176 ± 21 ms) and normal intrinsic PR interval (163 ± 26 ms). Of all the 63 patients, 22 (34.9%) were implanted with CRT‐pacemaker and the remaining received CRT‐defibrillator.

**TABLE 1:** Demographic data and baseline characteristics of the study population

	All patients	LVP	BVP	
	(*n* = 63)	(*n* = 34)	(*n* = 29)	p value
Age	63 ± 11	61 ± 12	65 ± 10	.23
Male (%)	45 (71.4%)	29 (85.3%)	16 (55.2%)	.01
Etiology				
Dilated Cardiomyopathy	42 (66.7%)	21 (61.8%)	21 (72.4%)	.37
Hypertension	21 (33.3%)	13 (38.2%)	8 (27.6%)	.37
Valvular heart disease	1 (1.6%)	1 (2.9%)	0 (0%)	1.00
Ischemic cardiomyopathy	2 (3.2%)	2 (5.9%)	0 (0%)	.50
NYHA class	2.7 ± 0.5	2.7 ± 0.4	2.6 ± 0.5	.34
Baseline QRS (ms)	176 ± 21	175 ± 24	177 ± 17	.77
Baseline PR (ms)	163 ± 26	164 ± 28	163 ± 25	.79^a^
LVEF (%)	27 ± 7	26 ± 7	28 ± 7	.20
LVEDD (mm)	69.3 ± 9.8	69.4 ± 8.9	69.3 ± 11.0	.98
LVESD (mm)	59.6 ± 10.6	59.6 ± 10.0	59.6 ± 11.5	.98
LVEDV (ml)	227.9 ± 79.7	227.5 ± 70.9	228.3 ± 91.1	.97
LVESV (ml)	168.4 ± 67.2	171.2 ± 64.5	164.7 ± 71.6	.71
6MWD (m)	341 ± 98	357 ± 103	323 ± 90	.18
Log [NT‐pro BNP (pg/ml)]	3.2 ± 0.5	3.2 ± 0.6	3.2 ± 0.4	.80
CRT‐P (%)	34.9	29.4	41.4	.32
Medications				
ACEI/ARB	47 (74.6%)	25 (73.5%)	22 (75.9%)	.83
ARNI	7 (11.1%)	4 (11.8%)	3 (10.3%)	.86
Beta‐blocker	53 (84.1%)	28 (82.4%)	25 (86.2%)	.68
Spironolactone	48 (76.2%)	24 (70.6%)	24 (82.8%)	.26
Digoxin	19 (30.2%)	11 (32.4%)	8 (27.6%)	.68

*Note:* Values are expressed as *n* or as mean ± standard deviation. p‐value: LVP group versus BVP group.

Abbreviations: 6MWD, 6‐minute walking distance; ACEI, angiotensin‐converting enzyme inhibitor; ARB, angiotensin II receptor blocker; ARNI, angiotensin receptor‐neprilysin inhibitor; CRT‐P(%), percentage of patients who were implanted with CRT‐pacemaker; LVEDD, left ventricular end‐diastolic dimension; LVEDV, left ventricular end‐diastolic volume; LVEF, left ventricular ejection fraction; LVESD, left ventricular end‐systolic dimension; LVESV, left ventricular end‐systolic volume; NT‐pro BNP, N‐terminal pro‐brain natriuretic peptide; NYHA class, New York Heart Association class.

^a^ p value by Mann–Whitney U test of baseline PR.

### 3.2 Clinical outcomes at 3‐ and 6‐month follow‐up

**FIGURE 2:**
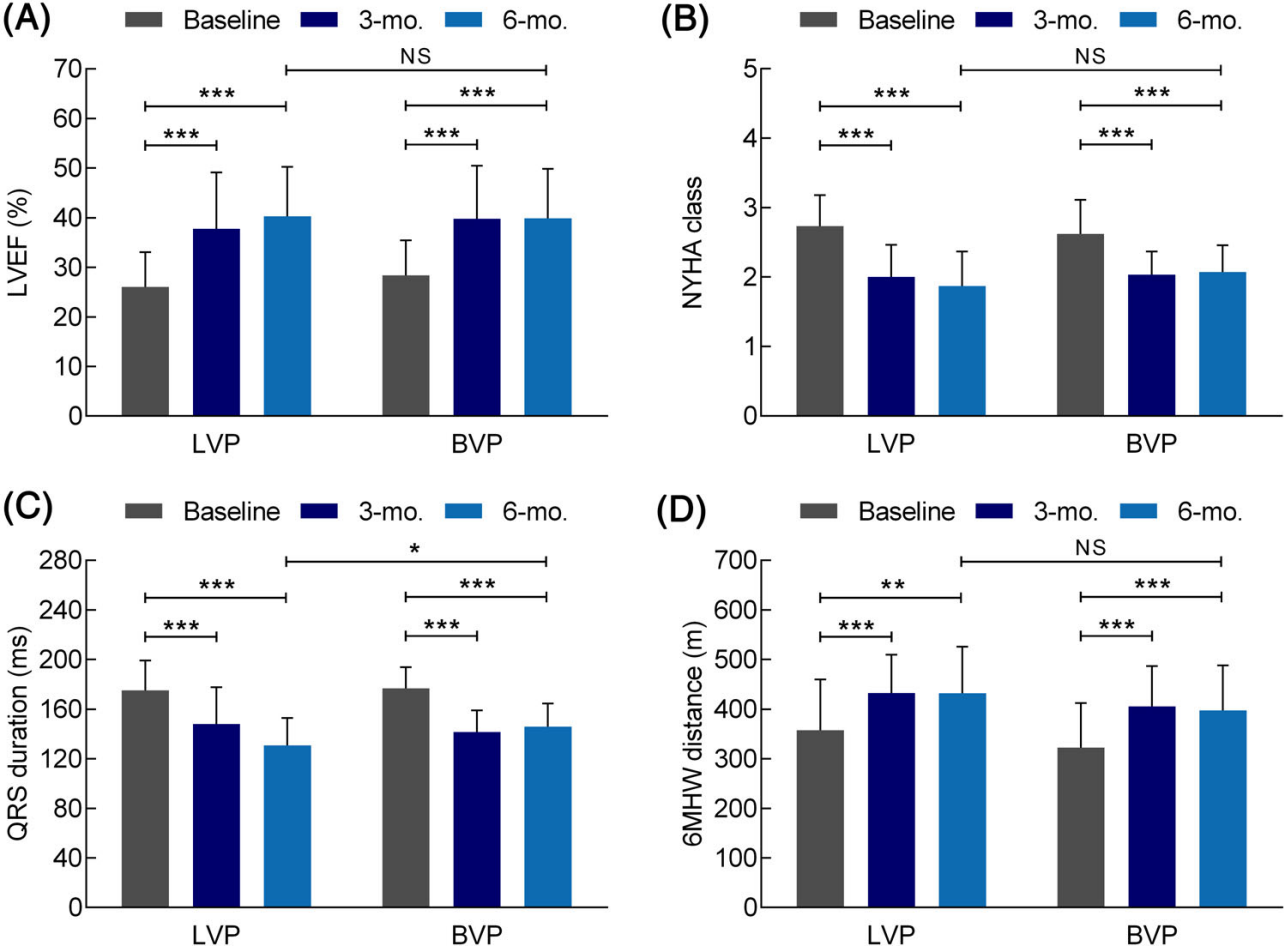
Clinical outcome in terms of left ventricular ejection fraction (LVEF) (A), NYHA class (B), QRS duration (C) and 6 min walking distance (6MWD) (D) at 3‐ and 6‐month follow‐up. ***, p < .001; **, p < .01; *, p < .05; NS, not significant. Sample size: LV‐only fusion pacing (LVP) group, baseline and 3 month *n* = 34, 6 month *n* = 31; biventricular pacing (BVP) group, baseline and 3 month *n* = 29, 6 month *n* = 27

Key echocardiographic and clinical endpoints were performed at baseline and 3‐/6‐month follow‐ups. In terms of LVEF, NYHA class and 6MWD, both groups had significant improvements at 3 months (p < .001) and 6 months (p < .001). Meanwhile, comparable results were observed between two groups at both 3‐month (p = .50) and 6‐month (p = .88) follow‐ups (Figure 2 and Table 2). Specifically, at 3 months, QRSd significantly reduced in both groups compared to baseline (LVP: 148 ± 30 ms vs. 175 ± 24 ms, p < .001; BVP: 141 ± 18 ms vs. 177 ± 17 ms, p < .001) with no significant between‐group difference (p = .36). At 6 months, however, QRSd in LVP group was found significantly smaller than that in BVP group (LVP: 131 ± 22 ms vs. BVP: 146 ± 19 ms, p < .05). LVP provided a trend of better CRT response rate at 6 months compared with BVP in terms of absolute LVEF increase larger than 10% (LVP: 71.0% vs. BVP: 61.5%, p = .45), LVESV decrease larger than 15% (LVP: 83.3% vs. BVP: 68.2%, p = .31), and NYHA class improved by at least one level (LVP: 77.4% vs. BVP: 59.3%, p = .14).

**TABLE 2:** Key echocardiographic and cardiac functional assessments at 3‐/6‐month follow‐up

	LVP		BVP		
	Mean ± SD	†p	Mean ± SD	†p	*p
NYHA class					
3 months	2.0 ± 0.5	<.01	2.0 ± 0.3	<.01	.74
6 months	1.9 ± 0.5	<.01	2.1 ± 0.4	<.01	.10
6MWD (m)					
3 months	433 ± 77	<.01	406 ± 81	<.01	.24
6 months	432 ± 94	<.01	397 ± 90	<.01	.18
QRS duration (ms)					
3 months	148 ± 30	<.01	141 ± 18	<.01	.36
6 months	131 ± 22	<.01	146 ± 19	<.01	.01
LVEDD (mm)					
3 months	67.2 ± 10.5	.03	64.5 ± 11.9	<.01	.37
6 months	66.8 ± 12.1	.08	62.2 ± 11.1	<.01	.15
LVESD (mm)					
3 months	55.7 ± 13.4	.02	53.6 ± 13.3	<.01	.57
6 months	55.2 ± 14.4	.07	49.6 ± 12.3	<.01	.14
LVEDV (ml)					
3 months	191.1 ± 80.5	<.01	187.1 ± 83.3	<.01	.86
6 months	187.6 ± 84.1	<.01‡‡	166.5 ± 89.7	<.01‡‡	.25‡
LVESV (ml)					
3 months	122.8 ± 70.4	<.01	119.7 ± 63.0	<.01	.87
6 months	113.3 ± 68.0	<.01‡‡	105.0 ± 65.2	<.01‡‡	.53‡
LVEF (%)					
3 months	38 ± 11	<.01	40 ± 11	<0.01	0.50
6 months	40 ± 10	<.01	40 ± 10	<0.01	0.88

*Note:* Sample size: LVP group, baseline and 3 month *n* = 34, 6‐month *n* = 31; BVP group, baseline and 3 month *n* = 29, 6 month *n* = 27.

* p‐value of between‐group difference; †p paired difference between baseline and 3‐/6‐month follow‐up; ‡p value from Mann–Whitney *U* test; ‡‡p value from Wilcoxon signed‐rank test.

Abbreviations: 6MWD, 6‐minute walking distance; BVP, biventricular pacing; LVEDD, left ventricular end‐diastolic dimension; LVEDV, left ventricular end‐diastolic volume; LVEF, left ventricular ejection fraction; LVESD, left ventricular end‐systolic dimension; LVESV, left ventricular end‐systolic volume; LVP, LV‐only fusion pacing; NYHA, New York Heart Association.

### 3.3 LVP subgroup analysis

In this study, the AdaptivCRT algorithm was enabled in LVP group to avoid RV pacing. Of 31 patients in LVP group, 25 had a high percentage of adaptive LV pacing that well fused with intrinsic RV activation (high‐aLVP% subgroup; average LV‐pacing percentage = 88.7%, average total ventricular pacing percentage = 96.1%) within 6 months. On the contrary, the remaining six patients had an average BVP percentage = 88.5% (average total ventricular pacing percentage = 95.5%) because AdaptivCRT algorithm could automatically switch the pacing mode to BVP when intrinsic RV conduction was failed to sense within default periodical check. Given that RV pacing output was set to the minimum for nonfunctional pacing, these six patients received LV‐only pacing without fusion with intrinsic RV activation (low‐aLVP% subgroup).

**FIGURE 3:**
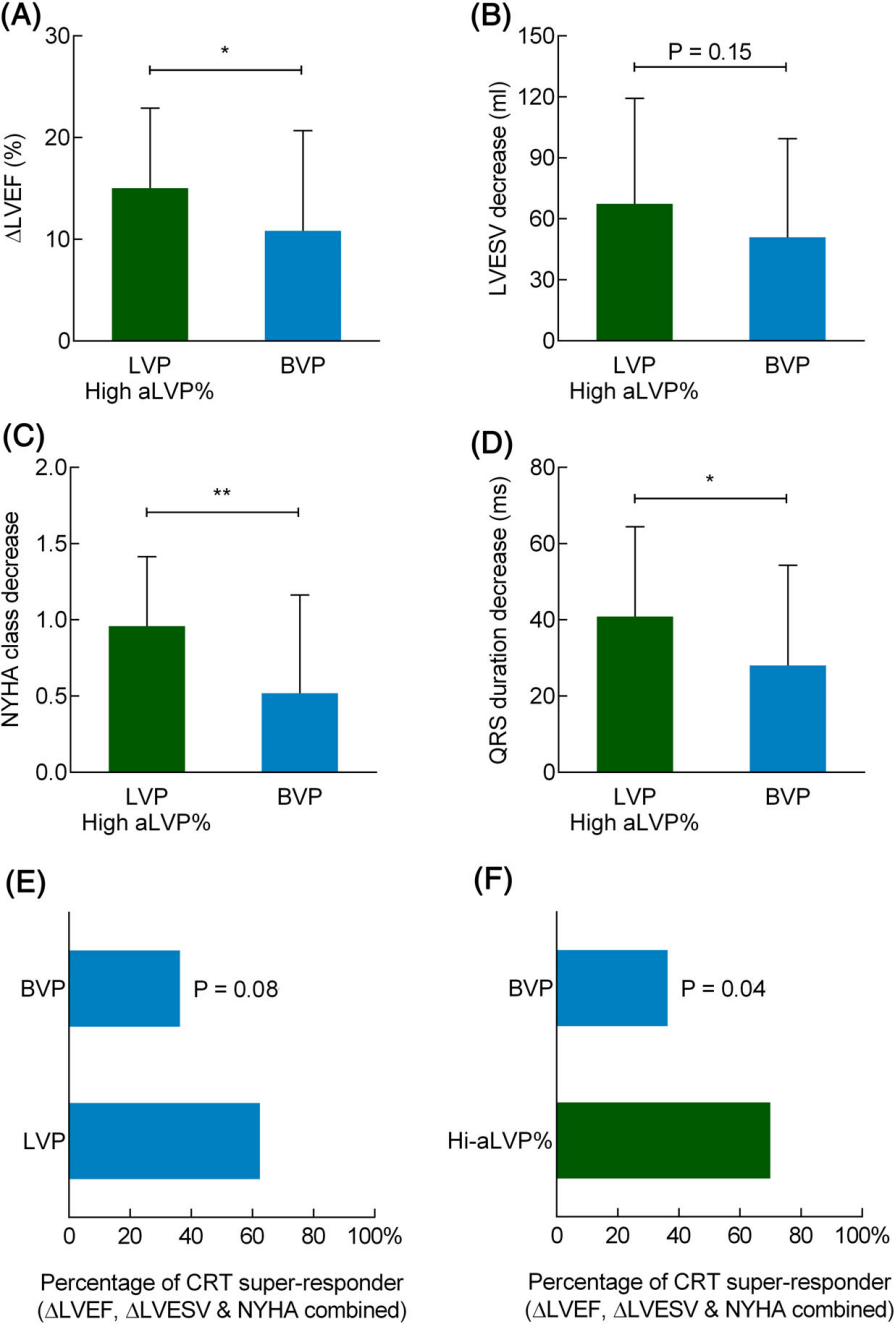
Clinical outcomes of the high‐aLVP% subgroup showing significantly greater clinical improvements in left ventricular ejection fraction (LVEF) (A), New York Heart Association (NYHA) (C) and QRS duration (QRSd) (D) at 6 months and a trend of more reduction in left ventricular end‐systolic volume (LVESV) (B) at 6 months, compared to the biventricular pacing (BVP) group. **, p < .01; *, p < .05. CRT super response rate at 6‐month follow‐up. Panel E: LVP group shows a trend of larger CRT super response compared with BVP. Panel F: High percentage of adaptive LVP is significantly associated with greater CRT super response compared with the BVP group. Sample size: LVP group, *n* = 31; High aLVP% subgroup, *n* = 25; BVP group, *n* = 27

Subgroup analysis revealed significant improvement of LVEF and LVESV at 6 months in both subgroups, compared to baseline, respectively. However, significant improvements in QRSd, NYHA class, and 6MWD were observed in high‐aLVP% at 6 months, but not in low‐aLVP% subgroup. Furthermore, comparing to the BVP group, the high‐aLVP% subgroup had significantly greater improvements in LVEF, NYHA (both p < .01) and QRSd (p < .05) at 6 months and a trend of better decrease in LVESV (p = .15; Figure 3), while no comparable improvements were observed in the low‐aLVP% subgroup compared to the BVP group (LVEF increase 11.3% ± 6.9% vs. 10.8% ± 9.8%, p = .46; LVESV decrease 47.2 ± 27.3 ml vs. 50.9 ± 48.5 ml, p = .44; NYHA decrease: 0.3 ± 0.5 vs. 0.5 ± 0.6, p = .26; QRSd shortening 44.0 ± 36.9 ms vs. 28.0 ± 26.3, p = .13).

With applying the definition of CRT super response, a trend of higher CRT super response rate was found in LVP group at 6 months compared with BVP group though statistical significance was not identified (p = .08, Figure 3(E)). However, the high‐aLVP% subgroup resulted in a significantly larger CRT super response rate than the BVP group (p = .04, Figure 3(F)).

## 4 DISCUSSION

The results of the present study demonstrated that LVP provides significantly improved outcomes in cardiac functional and echocardiographic measurements. The improvements were comparable with conventional echocardiography‐optimized BVP. Furthermore, a significantly higher CRT super‐response rate was identified in patients with a high percentage of LVP fused with RV activation compared with conventional BVP.

Findings in the present study are consistent with earlier studies that showed LVP with fusion and the avoidance of RV pacing had superior outcomes in hemodynamics and cardiac functions for a subset of patients with intact RV conduction.^3^, ^13^, ^14^ Different from prior studies^15^ in which high‐percentage (≥80%) of fused LVP was only accomplished in a much smaller percentage of patients, in the present study where RV pacing was not functioning, the superb clinical efficacy in high‐aLVP% subgroup further demonstrated the significance of the fusion of LVP and intact RV conduction. The underlying mechanism is the recruitment of natural RV conduction during LVP, making a more physiological pacing. A dynamically optimized AV and VV timing adapt AV and VV activation sequence according to varying physiological conditions and daily needs. On the contrary, conventional BVP resynchronizes right and left ventricles without the consideration of natural activation sequence in interventricular septum and RV. Moreover, the conventional BVP does not adjust AV timing to the optimum. Therefore, the CRT super‐response rate in patients with high‐LVP% was significantly higher than those with conventional BVP in the present study.

The AdaptivCRT algorithm^7^ was developed to support the continuous optimization of CRT intervals in order to take advantage of the potential superiority of LV fusion pacing in patients with normal RV activation. The primary results of the multicenter trials of AdaptivCRT demonstrated the non‐inferiority to echocardiographically optimized BVP in terms of clinical composite score and aortic outflow velocity time integral.^8^, ^9^, ^10^ Findings in these prior studies indicated that a higher CRT super response rate was found in patients with a high LVP percentage. The present study further showed that a high LVP percentage provided significantly greater benefits in LVEF increase, NYHA class improvement, QRSd decrease than conventional BVP, and a trend of larger LVESV decrease. These findings were consistent with a previous study which demonstrated that a high percentage of synchronized AdaptivCRT yielded better clinical outcomes than conventional BVP.^10^

Six patients in the present study experienced low‐percentage adaptive LVP during the follow‐up due to a prolonged sensed or paced AV interval likely with the progression of conduction delay in AV node (two out of six) or device detected non‐sustained ventricular/supraventricular tachycardia episodes (four out of six), letting the AdaptivCRT algorithm automatically switched to BVP mode. Heart failure related medication remains unchanged for these patients during follow‐up. As there was no functional RV pacing, these patients still had LV‐only pacing but without fusion with natural RV activation. Comparable improvements were observed in these six patients compared to the BVP group. Given the significant improvement of clinical outcomes in high‐aLVP% subgroup, the hypothesis was confirmed that LVP was at least equivalent to BVP. This was consistent with earlier studies showing no differences in major clinical outcome measures, including LVEF, NYHA class and 6MWD, between LVP and BVP.^5^, ^16^ More research of LV‐only pacing in patients with long PR interval is warranted.

### 4.1 Clinical perspectives

This study is the first prospective randomized multicenter clinical study to assess the feasibility of LV‐only pacing fused with intrinsic RV activation in CRT‐indicated patients. The favorable clinical results by LVP are comparable to conventional BVP and even better in patients with a high percentage of adaptive LVP. This study provided clinical evidence for a novel pacing mode, that is, LVP with an adaptive algorithm to optimize the fusion by continuously adjusting LV pacing timing to leverage intrinsic RV conduction and achieve dynamic and physiologic pacing. The positive results raise the possibility that cardiac resynchronization could be achieved by a novel two‐lead pacemaker system, that is, the right atrial pacing lead and LV epicardial pacing lead. In order to create a two‐lead LV‐only pacing system with the novel LV fusion pacing approach, the pacing lead in the right atrium and/or LV epicardium would be capable of sensing the RV activation, which would require a new type of sensing configuration. Furthermore, LVP may increase device longevity due to no RV pacing compared to BVP, which is in line with prior research.^5^ Moreover, obviating RV lead implantation can reduce the chance of tricuspid valve damage or regurgitation. However, defibrillation may not be applicable for this novel pacing approach at current stage.

### 4.2 Study limitations

The present study has some limitations. First, this pacing approach is not suitable for CRT‐indicated patients without LBBB or persistent atrial fibrillation. Defibrillation is not applicable for this pacing approach. In addition, LV‐only pacing without fusion that leads to less improvement in cardiac function might be a result of prolonged intrinsic PR interval or ventricular/supraventricular tachycardia. Therefore, more research is warranted to investigate adaptive LVP in these status. Meanwhile, the sample size of patients enrolled in this feasibility study is relatively small. Longer follow‐up is warranted to better assess hospitalization and mortality of adaptive LVP compared to conventional BVP.

## 5 CONCLUSIONS

The present study demonstrated comparable clinical outcomes between adaptive LVP group and conventional BVP group. Moreover, our study found that high‐percentage adaptive LVP was significantly associated with better clinical outcomes and a higher CRT super response rate than BVP. This finding raises the possibility that an adaptive LVP algorithm with appropriate RV sensing to fuse with intrinsic RV activation in a two‐lead (right atrium and LV) device may provide clinical benefit in a subset of CRT patients with intact AV conduction.

## CONFLICT OF INTEREST

Yangang Su, Wei Hua, Farong Shen, Jiangang Zou, Baopeng Tang, and Keping Chen have received research grants from Medtronic. Shu Zhang has received consulting fees from Boston Scientific, Medtronic, St. Jude Medical, and Biotronik. Xiaohong Zhou, Xue Zhang and Hongyang Lu are employees of Medtronic. Yixiu Liang and Lang He have no competing interest to disclose.

## Data Availability

On reasonable request, the datasets used and/or analyzed of the study will be available from the corresponding author.

## References

[1] Abraham WT, Fisher WG, Smith AL, et al. Cardiac resynchronization in chronic heart failure. N Engl J Med. 2002;346(24):1845‐1853. 12063368 10.1056/NEJMoa013168

[2] Young JB, Abraham WT, Smith AL, et al. Combined cardiac resynchronization and implantable cardioversion defibrillation in advanced chronic heart failure: the MIRACLE ICD trial. Jama. 2003;289(20):2685‐2694. 12771115 10.1001/jama.289.20.2685

[3] Van Gelder BM, Bracke FA, Meijer A, et al. The hemodynamic effect of intrinsic conduction during left ventricular pacing as compared to biventricular pacing. J Am Coll Cardiol. 2005;46(12):2305‐2310. 16360063 10.1016/j.jacc.2005.02.098

[4] Rao RK, Kumar UN, Schafer J, Viloria E, de Lurgio D, Foster E. Reduced ventricular volumes and improved systolic function with cardiac resynchronization therapy: a randomized trial comparing simultaneous biventricular pacing, sequential biventricular pacing, and left ventricular pacing. Circulation. 2007;115(16):2136‐2144. 17420340 10.1161/CIRCULATIONAHA.106.634444

[5] Boriani G, Kranig W, Donal E, et al. A randomized double‐blind comparison of biventricular versus left ventricular stimulation for cardiac resynchronization therapy: the biventricular versus left Univentricular pacing with ICD Back‐up in heart failure patients (B‐LEFT HF) trial. Am Heart J. 2010;159(6):1052‐1058. 20569719 10.1016/j.ahj.2010.03.008

[6] Thibault B, Ducharme A, Harel F, et al. Left ventricular versus simultaneous biventricular pacing in patients with heart failure and a QRS complex≥ 120 milliseconds. Circulation. 2011;124(25):2874‐2881. 22104549 10.1161/CIRCULATIONAHA.111.032904

[7] Krum H, Lemke B, Birnie D, et al. A novel algorithm for individualized cardiac resynchronization therapy: rationale and design of the adaptive cardiac resynchronization therapy trial. Am Heart J. 2012;163(5):747‐752. 22607850 10.1016/j.ahj.2012.02.007

[8] Martin DO, Lemke B, Birnie D, et al. Investigation of a novel algorithm for synchronized left‐ventricular pacing and ambulatory optimization of cardiac resynchronization therapy: results of the adaptive CRT trial. Heart Rhythm. 2012;9(11):1807‐1814. 22796472 10.1016/j.hrthm.2012.07.009

[9] Birnie D, Lemke B, Aonuma K, et al. Clinical outcomes with synchronized left ventricular pacing: analysis of the adaptive CRT trial. Heart Rhythm. 2013;10(9):1368‐1374. 23851059 10.1016/j.hrthm.2013.07.007

[10] Birnie D, Hudnall H, Lemke B, et al. Continuous optimization of cardiac resynchronization therapy reduces atrial fibrillation in heart failure patients: results of the adaptive cardiac resynchronization therapy trial. Heart Rhythm. 2017;14(12):1820‐1825. 28893549 10.1016/j.hrthm.2017.08.017

[11] Gorcsan J III, Abraham T, Agler DA, et al. Echocardiography for cardiac resynchronization therapy: recommendations for performance and reporting–a report from the American Society of Echocardiography Dyssynchrony writing group endorsed by the Heart Rhythm Society. J Am Soc Echocardiogr. 2008;21(3):191‐213. 18314047 10.1016/j.echo.2008.01.003

[12] Antonio N, Teixeira R, Coelho L, et al. Identification of 'super‐responders' to cardiac resynchronization therapy: the importance of symptom duration and left ventricular geometry. Europace. 2009;11(3):343‐349. 19240109 10.1093/europace/eup038

[13] Kass DA, Chen CH, Curry C, et al. Improved left ventricular mechanics from acute VDD pacing in patients with dilated cardiomyopathy and ventricular conduction delay. Circulation. 1999;99(12):1567‐1573. 10096932 10.1161/01.cir.99.12.1567

[14] Lee KL, Burnes JE, Mullen TJ, et al. Avoidance of right ventricular pacing in cardiac resynchronization therapy improves right ventricular hemodynamics in heart failure patients. J Cardiovasc Electrophysiol. 2007;18(5):497‐504. 17428272 10.1111/j.1540-8167.2007.00788.x

[15] Burns KV, Gage RM, Curtin AE, Gorcsan J III, Bank AJ. Left ventricular‐only pacing in heart failure patients with normal atrioventricular conduction improves global function and left ventricular regional mechanics compared with biventricular pacing: an adaptive cardiac resynchronization therapy sub‐study. Eur J Heart Fail. 2017;19(10):1335‐1343. 28653458 10.1002/ejhf.906

[16] Sirker A, Thomas M, Baker S, et al. Cardiac resynchronization therapy: left or left‐and‐right for optimal symptomatic effect—the LOLA ROSE study. Europace. 2007;9(10):862‐868. 17684066 10.1093/europace/eum161

